# Adverse outcomes after colposcopy

**DOI:** 10.1186/1472-6874-11-2

**Published:** 2011-01-20

**Authors:** Sarah M Flanagan, Sue Wilson, David Luesley, Sarah L Damery, Sheila M Greenfield

**Affiliations:** 1 Department of Primary Care Clinical Sciences, The University of Birmingham, Edgbaston, Birmingham, B15 2TT, UK

## Abstract

**Background:**

Colposcopy is an essential part of the National Health Service Cervical Screening Programme (NHSCSP). It is used for both diagnosis and treatment of pre-cancerous cells of the cervix.

Despite colposcopy being a commonly performed and relatively invasive procedure, very little research has explored the potential long-term impacts of colposcopic examination upon patient quality of life.

The aim of this study is to investigate and quantify any potential reduction in women's quality of life following a colposcopy procedure. More specifically, the degree of female sexual dysfunction and the excess risk of adverse events in those undergoing colposcopy will be explored. If such risks are identified, these can be communicated to women before undergoing colposcopy. It will also assist in identifying whether there are particular sub-groups at greater risk and if so, this may lead to a re-evaluation of current recommendations concerning colposcopically directed treatments.

**Methods/design:**

Cohort study using postal surveys to assess sexual function and quality of life in women who have attended for colposcopy (cases), compared with those who have not attended colposcopy (controls). The prevalence and excess risk of female sexual dysfunction will be determined. Logistic regression will identify the predictors of adverse outcomes.

**Discussion:**

There are more than 400,000 colposcopy appointments each year in England, of which 134,000 are new referrals. There is some evidence that there may be long-term implications for women treated under colposcopy with respect to adverse obstetric outcomes, persisting anxiety, increased rates of sexual dysfunction and reduced quality of life. Reliably establishing whether such adverse outcomes exist and the excess risk of adverse events will facilitate informed decision-making and patient choice.

## Background

Colpsocopy is an essential part of the National Health Service Cervical Screening Programme (NHSCSP). It is a detailed examination of the cervix, performed by a doctor or qualified colposcopist in an outpatient setting. Colposcopy clinics enable the diagnosis and treatment of Cervical Intraepithelial Neoplasia (CIN) [1]. Women are invited for colposcopy following a referral generated via the NHSCSP recommending further investigation of potential cell abnormalities discovered during routine cervical screening or if they are experiencing symptoms that warrant further investigation.

There are more than 400,000 colposcopy appointments each year, of which 134,000 are new referrals [2]. Colposcopy is therefore a relatively common procedure, and has a treatment success rate exceeding [3] 90%. There are typically few complications [4,5]. Very little research has explored the long-term consequences of colposcopic investigation, other than with respect to recurrence of CIN [6], the incidence of cervical stenosis, [7] and the impact on subsequent pregnancies or recurrence [8-11].

The long-term physical and psychological consequences of invasive cancer are well described [12]. Psychological well-being scores amongst cancer patients remain lower than those of patients with other chronic illnesses or healthy subjects irrespective of age, cancer site, or stage of disease; and the provision of psychological interventions for patients facing cancer treatment has been recommended as an integral component of cancer management [2]. Furthermore, studies exploring the quality of life of cancer survivors suggest that fear of the diagnostic process can be as traumatic as the effects of cancer treatment. Longitudinal studies assessing the consequences of hysterectomy in the treatment of early stage cervical carcinoma report that the majority of women (>90%) report one or more symptoms associated with reduced quality of life and a persistent negative impact on sexual interest has been reported [13,14]. Jensen et al utilised a validated self-assessment questionnaire (the Sexual function-Vaginal changes Questionnaire - SVQ) and found that radical hysterectomy was associated with a long-term reduction in patients' sexual interest [14].

There is a considerable body of evidence relating to the anxiety and distress associated with an abnormal smear, attending a colposcopy appointment, and the diagnosis of CIN or cancer [2,13,15]. Almost all of the published literature reports an adverse impact on anxiety and quality of life associated with these clinical investigations and diagnoses. Diagnosis of CIN has been associated with a perceived threat to life and/or fertility, feelings of anger and resentment [16-18], and negative impacts upon body image and sexual functioning [19]. Diagnosis can also have a stigmatising effect [20], which may lead women to avoid subsequent screening [21-23]. Studies have found that the main reason for non-attendance for cervical screening is patients' belief that it is a test for cancer alone, and fear of a positive result [21-23].

Treatment of CIN has an adverse effect on subsequent fertility and obstetric outcomes [7-9]. There is some evidence that women who have undergone colposcopy report adverse effects upon their sex life [1,16-18,24-26]. Women have been reported to experience impaired sexual functioning after colposcopy [19,27]. Posner and Vessey study found that 14% of women reported that their sex life was not 'back to normal' six to nine months after colposcopy and that 19% of women's sex lives were adversely affected subsequent to treatment [13].

Despite qualitative research suggesting the possibility of long-term adverse consequences of colposcopy [13], research has been undertaken to determine the generalisability of these findings or to quantify the prevalence of these adverse impacts. Quantitative studies have to date been small-scale, underpowered, and tend to be restricted to the time of the abnormal smear or colposcopy [19,27]. Furthermore, none have been of sufficient size to differentiate between women who underwent colposcopy purely for diagnosis and those who had treatment. The need to establish the long-term psychological consequences of colposcopy has been acknowledged [18], and a study of sufficient size and follow-up is required.

Unanswered questions relate to: i) whether there is a reduction in quality of life and an excess of female sexual dysfunction (FSD) subsequent to colposcopy; ii) the extent of any excess risk, and iii) whether the potential adverse impacts following colposcopy differ according to the level of intervention (diagnosis or treatment) which women undergo. This cohort study aims to determine the association between colposcopy and treatment for CIN and subsequent risk of sexual dysfunction and/or reduced quality of life.

### Aims and Objectives

#### Primary Aim

The primary aim of this cohort study is to determine the long-term impact of colposcopic intervention and treatment for CIN on quality of life (QoL) and sexual functioning.

#### Objectives

(1) To conduct a systematic review of the literature in relation to colposcopy and its effect upon quality of life (physical and psychological) including sexual functioning.

(2) To accurately establish the prevalence of sexual dysfunction subsequent to colposcopy.

(3) To identify any sub-groups of women who may be at increased risk of sexual dysfunction and reduced quality of life.

(4) To determine the need for interventions to reduce the long-term adverse impacts of colposcopy.

## Methods/design

### Summary of study design

#### Cohort study

A short postal questionnaire will estimate the prevalence of Female Sexual Dysfunction (FSD). A second, more detailed questionnaire will address other aspects of QoL. Postal questionnaires will be sent to women (n = 1050) who have attended for colposcopy (cases), and to age and deprivation matched controls (n = 1050) who have not attended colposcopy. The Index of Multiple Deprivation (IMD 2007) used as a proxy measure for deprivation [28].

### Eligibility criteria

Cases: New referrals - women aged 25 to 65 who have undergone colposcopy at least 12 months previously (between April 2008 and March 2009). Cases will be stratified by level of intervention.

1. Low risk - colposcopy only: no treatment or investigation (n = 350)

2. Medium risk - colposcopy with investigation/diagnosis i.e. punch biopsy (n = 350)

3. High risk - colposcopy and treatment i.e. loop excision, or cone biopsy (n = 350)

Controls: Women aged 25 to 65 who have never undergone colposcopy (n = 1050). Controls will be age (+/- 3 years) and deprivation score matched with controls.

### Exclusion criteria

Cases: Women who have undergone treatment which has not been colposcopically directed, for example women who have been treated by hysterectomy. Women with a previous/current diagnosis of invasive cancer.

Controls: Women who have undergone colposcopy or have been diagnosed with a gynaecological malignancy.

### Recruitment of participants

Study Population - Cases will be identified using the patient databases of five participating NHS Colposcopy units within the West Midlands. Up to four general practices will be recruited to identify control participants. Practices will be purposively recruited to ensure that controls can be matched by age and IMD 2007.

### Invitation method

Potential participants will receive a covering letter and a patient information leaflet (PIL) explaining the study in depth, with a questionnaire and freepost envelope for questionnaire return. Questionnaires will be marked with a unique identifier and one reminder will be sent to all non-responders.

Responders to the first questionnaire who indicate their willingness to participate in the next research phase will receive a subsequent more detailed questionnaire. To maximise uptake amongst control participants, we will provided information about how their participation will enable exploration of this under-researched area of women's health. A pilot/feasibility study has confirmed the acceptability of the data collection tools and patient information leaflet. Feedback from this has informed revisions of the PIL and questionnaire with a view to maximising participation.

### Data Collection tool

#### Questionnaire details

Initially, potential participants will receive a brief questionnaire to establish the prevalence of sexual problems using six questions used in the National Survey of Sexual Attitudes and Lifestyles (2000) [29] that relate to sexual functioning. It also includes four basic questions relating to general health and responders are asked to rate their satisfaction levels with sex life and relationships on a likert scales. It will also contain an open question with space provided for participants to provide any further comments. Participants will also be invited to consent to receive the second questionnaire.

The second questionnaire will collect more detailed demographic information and details of outcomes and the predictors of adverse outcomes (Table 1).

**Table 1:** Information collected in the second questionnaire

Outcomes	Quality of Life [30], sexual functioning [31], anxiety and depression [32], cervical stenosis, obstetric outcomes
Confounders	Socio-demographic factors: Age, ethnicity [33], deprivation [28], relationships, education
	Lifestyle factors: smoking, alcohol, problem drug use
	Health-related factors: obstetric history, history of sexually transmitted disease, number of visits to GP, physical health problems

The following validated measures will be used to measure sexual function, levels of anxiety and depression and quality of life:

Female Sexual Function Index (FSFI) which provides information about a woman's sexual functioning [31]. A score below 26.5 indicates sexual dysfunction.

The Hospital Anxiety and Depression (HADS) scale, which assesses levels of anxiety and depression. HADS has been found to perform well in assessing the symptom severity and caseness of anxiety disorders and depression in somatic, psychiatric and primary care patients as well as in the general population [32]. HADS contains 14 items, each of which is marked on a four-point sub-scale. Scores of 11 or more on either subscale are considered to indicate a significant 'case' of psychological morbidity, while scores of 8 to 10 represent a 'borderline' case, and 0 to 7 are 'normal' [34].

Quality of life will be measured using the WHOQOL-BREF (The World Health Quality of Life - BREF) incorporating physical health, psychological health, social relationships and environmental factors [30].

All control participants will receive the same questionnaires as the cases.

### Analysis

The prevalence of FSD will be standardised to the England and Wales population [35]. Multivariate analyses will describe patterns of differences between patients according to the level of investigation/treatment associated with their colposcopy (in terms of low, medium or high risk). FSD, anxiety and QoL will be compared for the three different groups (low, medium and high risk) and controls. Logistic regression analyses including socio-demographic variables (age, ethnicity, deprivation) and health-related factors (e.g. co-morbidities) will aim to identify the predictors of adverse outcomes (e.g. FSD).

Open comments will be analysed with the help of a computer-package (SPSS Text Analysis for Surveys™), by content analysis using both quantitative (e.g. number of times a word/phrase mentioned) and qualitative techniques (e.g. examples of participants' own words to reflect emerging themes) [36].

### Justification of sample size

**Figure 1:**
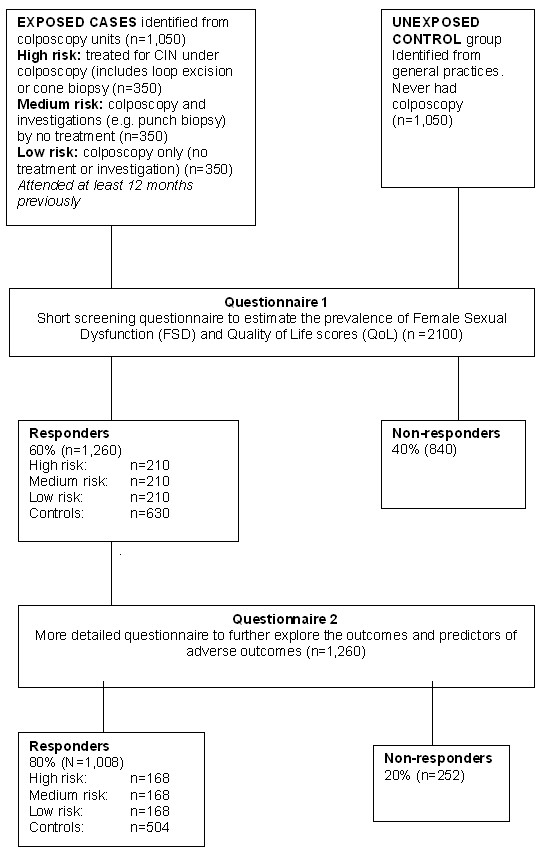
Study schematic

Each year, the five collaborating centres have 12,400 colposcopy appointments. 6693 individuals are estimated to be eligible for inclusion within this study (i.e. excluding follow-up, non-attendance, cancellation). Eligible women comprise those treated (11.5%), not requiring treatment (65%) and those having investigation (e.g. biopsy) but no further treatment (23.5%). Data have been based upon colposcopy records of three participating colposcopy units. The mean age at colposcopy is 38 years (range 17 to 82) and the background (no colposcopy) prevalence of FSD is assumed to be 15% [35]. To estimate a doubling in the risk of FSD (from 15 to 30%), 160 participants are required in each group (90% power, 5% significance). The smallest group (high risk) comprises 770 women per annum. Conservatively assuming a questionnaire response rate of 50% (we usually achieve >60% [37,38], the required sample size would be achieved through mailing 350 in each group. Assuming an average practice list size of 6,000, that 15% [39] are aged between 20 and 40 (most common age range for colposcopy), and that 70% have not experienced colposcopy, 630 patients per practice will be eligible as controls [Figure 1]. Four practices with variability in socio-demographic profiles will be recruited.

Practices will be identified via the Midlands Research Practices Consortium (MidReC). This consists of a network of over 600 practices, covering a representative population of over four million residents of the West Midlands [40].

### Ethical approvals

Ethics approval has been provided by The Black Country Research Ethics Committee on 15/03/10 (reference: 10/H1202/9).

R&D approval has been recieved from four R&D trusts: Heart of England NHS Trust, Birmingham Women's NHS Foundation Trust, Sandwell and West Birmingham NHS Trust and Worcestershire PCT.

## Discussion

Colposcopy is a relatively common procedure, with over 400,000 appointments attended each year. It is an essential part of the National Health Service Cervical Screening Programme and enables the diagnosis and treatment of pre-cancerous changes in the cervix. Although it is a relatively safe procedure and an effective treatment for CIN, previous studies have indicated that it can have adverse effects upon obstetric outcomes and may increase the incidence of cervical stenosis. Furthermore, evidence suggests that cervical screening, undergoing colposcopy and subsequent treatment for CIN may have adverse impacts upon women's psychological, physical health and sexual health and well-being. As these studies have been relatively small or based on qualitative data, a study of sufficient size is required to determine the long-term impacts of colposcopy. In addition, none of the current studies have been powered to differentiate between the impacts experienced by women that underwent colposcopy purely for diagnosis and those who have treatment. In this study, we will stratify cases depending upon whether patients were 'treated' at colposcopy, underwent investigation (biopsy) or had no treatment or investigation. This study will enable us to establish, for example, if patients who were 'treated' experienced adverse events to a greater extent than patients who underwent investigation alone.

It will also assist in identifying whether there are particular population sub-groups who are more likely to be at an increased risk of adverse effects after colposcopy. If such risks are identified, this may lead to a reconsideration of the benefits of earlier intervention and a re-evaluation of the current recommendations concerning colposcopically directed treatments [4]. Women who are currently at low risk of CIN may not be treated by colposcopy as promptly as current guidelines indicate. This may allow any potential abnormalities that may revert to normal to do so without the need for more invasive investigation. It may also be that this group of women can be monitored via cervical screening. Women at high risk of experiencing adverse outcomes could be monitored more closely and offered appropriate support and advice around potential adverse outcomes. If no excess risk is identified, we can reassure women and clinicians that there are no adverse outcomes associated with colposcopy in terms of psycho-sexual and general health and well-being.

### Ethical approvals

Ethics approval has been provided by The Black Country Research Ethics Committee on 15/03/10 (reference: 10/H1202/9).

R&D approval has been received from four R&D trusts: Heart of England NHS Trust, Birmingham Women's NHS Foundation Trust, Sandwell and West Birmingham NHS Trust and Worcestershire PCT.

## Competing interests

The authors declare that they have no competing interests.

## Authors' contributions

SW and DL conceived of the study and SW completed the original funding application upon which the protocol is based. SF wrote the updated version of the protocol manuscript and SD, SG and SW critically revised the manuscript manuscript. All authors read and approved the final manuscript.

## Pre-publication history

The pre-publication history for this paper can be accessed here:

http://www.biomedcentral.com/1472-6874/11/2/prepub
